# Overweight and Obesity among Children (10-13 years) in Bahrain:A comparison between Two International Standards

**DOI:** 10.12669/pjms.303.4796

**Published:** 2014

**Authors:** Abdulrahman O. Musaiger, Mariam Al-Mannai, Qazi Al-Marzog

**Affiliations:** 1 Abdulrahman O. Musaiger, Arab Center for Nutrition, Bahrain.; 2 Mariam Al-Mannai, Department of Mathematic, College of Science, University of Bahrain, Bahrain.; 3 Qazi Al-Marzog, Ministry of Education, Bahrain.

**Keywords:** Bahrain, Obesity, Overweight, School children

## Abstract

***Objective:*** Obesity has become one of the main public health problems worldwide. Childhood obesity rate is growing very fast in both developed and developing countries. This paper aimed to explore the prevalence of overweight and obesity among school children aged 10-13 years in Bahrain, and to find out the difference in this prevalence when using two international standards.

***Methods:*** A multistage stratified sampling procedure was used to select 2146 students (1068 males, 1078 females) from public schools in Bahrain. Weight and height were measured and Body Mass Index for age and sex was calculated to determine the obesity levels. Both International Obesity Task Force (IOTF) and World Health Organization (WHO) references were used to determine the prevalence of overweight and obesity.

***Results:*** The findings revealed that the prevalence of overweight and obesity ranged from 15.7% to 28.9% among males and from 21.1% to 30.7% among females. The WHO reference standard provided higher prevalence of overweight and obesity than IOTF reference.

***Conclusion:*** The study confirmed that obesity is a problem of concern in Bahraini school children and calls for intervention programme to combat obesity in schools. However, the standard used to determine obesity levels should be carefully selected and interpreted.

## INTRODUCTION

Childhood obesity has reached an alarming level in the Arab Gulf states, including Bahrain. The prevalence of overweight and obesity among school children (14-18 years) in these countries ranged from 30% to 45%.^1^ It is well documented that obesity during childhood is a risk factor for establishing obesity and its related chronic diseases during adulthood, such as cardiovascular disease, diabetes mellitus, hypertension and some type of cancer.^2^ Such diseases are representing more than 60% of annual morbidity and mortality in the Arab Gulf countries. The nutrition transition, which was happened in most Arab countries during the past three decades play an important role in changing dietary habits, lifestyle and socio-economic status in these countries. The results are high proportion of obesity among both children and adults.^3^ Therefore, controlling obesity especially during childhood will decrease the health burden of these diseases. 

Previous studies on obesity, which were carried out on school children in Bahrain were based on a small sample size for each age group, and focused on age 15-18 years^4^ or 12-18 years^5^ and one study covered 6-18 years.^6^ However, most of these studies used CDC reference standard^6^ or NHANES standard.^4^ Nowadays these two standards are rarely recommended for measuring obesity among children outside USA, as International Obesity Taskforce (IOTF)^7^ and World Health Organization (WHO)^8^ have developed international standards to be used in countries who did not have their local growth standards, and also used for the sake of comparison in prevalence of obesity between different countries.

Health authorities in Bahrain along with other Arab Gulf countries have become more aware about the health and economic burden of obesity and planning to develop plan of action to prevent and control of obesity. Such plan should focus on prevention of obesity during childhood. The need for comprehensive data on the prevalence of overweight and obesity is essential for planning of any intervention programme to combat obesity in school children in Bahrain. Therefore, the aim of this study was to provide sufficient information on obesity among school children aged 10-13 years in Bahrain; using two commonly used reference standards; IOTF and WHO standards.

## METHODS

Data of this study were extracted from the national survey on food habits and lifestyle of primary school children in Bahrain.^9^ The target children were those enrolled in 4-6 levels of education. A multistage stratified random sampling method was used to select the children. Bahrain is composed of five governorates. The schools were divided into boys and girls primary schools, and selected proportionally from each governorate using simple random procedure. Then, the classes were selected from each primary levels (levels 4, 5 and 6), in each school using simple random method. The study was ethically approval by both the Arab Center for Nutrition, Bahrain and the Research Committee at the Ministry of Education, Bahrain. The total sample size was 2146 students (1068 males and 1078 females). Detailed information on distribution of the sample by age and gender is given in Table-I.

Weight and height were taken using a standard procedure by physical education teachers. All the measurements were taken with minimal clothing and without shoes. The International Obesity Task Force (IOTF)^7^ and the World Health Organization^8^ reference standards were used to classify the children to non-overweight, overweight and obese using Body Mass Index cut-offs by age and gender.

**Table-I:** Sample size of Bahraini school children (10-13 years) by age and gender

*Age (years)*	*Male*		*Female*		*Total*	
	*No.*	*%*	*No.*	*%*	*No.*	*%*
10-10.9	241	57.8	176	42.2	417	100.0
11-11.9	350	49.6	356	50.4	706	100.0
12-12.9	363	44.6	451	55.4	814	100.0
13-13.9	114	54.5	95	45.5	209	100.0
**Total**	1068	49.8	1078	50.2	2146	100.0

**Table-II:** Prevalence of non- overweight, overweight and obesity among school children (10-13 years) in Bahraini using IOTF and WHO reference standards

*Age (years)*	*Reference*	*Non-overweight (%)*		*Overweight (%)*		*Obese (%)*	
		*M*	*F*	*M*	*F*	*M*	*F*
10-10.9	IOTF	80.9	80.1	12.4	9.7	6.6	10.2
	WHO	73.0	73.9	13.3	14.8	13.7	11.4
11-11.9	IOTF	77.4	76.1	14.6	16.9	8.0	7.0
	WHO	70.6	69.4	14.6	19.7	14.9	11.0
12-12.9	IOTF	80.4	77.4	12.9	14.2	6.6	8.4
	WHO	71.1	71.2	18.7	16.2	10.2	12.6
13-13.9	IOTF	84.2	78.9	6.1	13.7	9.6	7.4
	WHO	82.5	73.7	6.1	15.8	11.4	10.5

## RESULTS

The distribution of studied school children by age and gender is shown in Table-I. The proportion of males was higher at age 10 and 13 years; whereas the proportion of females exceeded males at age 12 year. The proportion at age 11 year was almost equal (49.6% and 50.4%, for males and females, respectively).

 Prevalence of non-overweight, overweight and obesity among school children aged 10 to 13 years, using IOTF and WHO reference standards are presented in Table-II. In general, overweight and obesity ranged from 15.7% to 28.9% among males; and 21.1% to 30.7% among females. The WHO reference standard provided higher prevalence of both overweight and obesity than IOTF reference standard. Using IOTF reference, males were less likely to have overweight than females at all ages, except at age 10 year, whereas for obesity males at ages 10 and 12 had lower proportion than females and the opposite at ages 11 and 13 years. Using WHO reference standard, males were more prone to have obesity than females, except at age 11 year, where females had higher prevalence of obesity (10.2% and 12.6%, for males and females, respectively).

## DISCUSSION

This study suggests a high prevalence of overweight and obesity among Bahraini children aged 10-13 years. Comparing the proportion of overweight and obesity among children aged **12 to **13 years with those reported previously^5^ using same standard (IOTF), we can notice that our study provide lower prevalence of overweight and obesity. This could be attributed to the very small sample size for each age group in the previous study. However, when we compared the prevalence of obesity among the children in this study with those reported among 1-5 years old^10^, we can observe a sharp increase in obesity. The proportion of overweight and obesity in Bahraini children aged 1-5 years old was 15% and 18% in males and females, respectively, and it reached 25% and 28% in males and females, at age 10-13 years old in this study, respectively. However, this finding is a rough indicator rather than absolute trends, as the two studies were cross-sectional and did not deal with the same children.

Several factors could have contributed to high prevalence of obesity among Bahraini children, such as high intake of energy density foods, sedentary lifestyle and inactivity. The dietary habits of school children in Bahrain characterized by high consumption of fast food, sweet and chocolates and sugary beverages, and low consumption of fruit and vegetables, and milk.^11^ There is good evidence that high consumption of fast food^12^ and sugary beverages^13^ have positive association with obesity. Furthermore, the frequent intakes of fruit and vegetables^14^ and milk products^15^ have shown to have negative association with obesity. Sedentary lifestyle has increased markedly among Bahraini school children, with long viewing of television, long use of video games and internet.^11^ Gharib and Rasheed^6^ showed that non-overweight school children in Bahrain were less likely to watch television per week and less using electronic-computer games per week, than overweight and obese children.

Previous studies in Bahrain found that girls were less prone to practice exercise than boys, especially out of school.^6^^,^^11^ This may indicate that girls are more at risk of obesity at adulthood. Data from Bahraini Ministry of Health indicate that 67% of men and 71% of women were overweight and obese.^16^ Therefore it is important to promote physical activity and healthy eating at school age. However, girls in the Arab World in general are facing many socio-cultural factors and male-biased related to practicing physical activity. For example, most of public sport facilities are male oriented and very few available to females. Most girls and women cannot practice exercise outdoor and with sport dress because of cultural or religious reasons. In Bahrain, the majority of girls or women who are allowed by their families to practice exercise outdoor, do such with traditional dress, which is not comfortable for exercise purpose.^1^

The obesity epidemic environment in Bahrain, especially in school children and youth creates the need for establishing an intervention programme to prevent and control of obesity among these age groups. The standard used to determine the overweight and obesity prevalence should be carefully selected, as these standards do not give same findings. The current data of this study provide additional information, which may be used in planning appropriate intervention programme, which should consider promoting healthy eating habits and physical activity.

## Authors’ Contribution:

**AOM and QA:** Participated in conception and design the study. **QA:** Supervised the data collection. **MA:** Did the statistical analysis. **AOM:** Drafted the manuscript. All authors approved the final version of the manuscript.
